# Sex-Based Differences in Rotational Atherectomy and Long-Term Clinical Outcomes

**DOI:** 10.3390/jcm12155044

**Published:** 2023-07-31

**Authors:** Mohamed Ayoub, Selina Lutsch, Michael Behnes, Muharrem Akin, Tobias Schupp, Ibrahim Akin, Volker Rudolph, Dirk Westermann, Kambis Mashayekhi

**Affiliations:** 1 Division of Cardiology and Angiology, Heart Center University of Bochum, 32545 Bad Oeynhausen, Germany; s.lutsch@hdz-nrw.de (S.L.);; 2 Department of Cardiology, Angiology, Haemostaseology and Medical Intensive Care, University Medical Centre Mannheim, Medical Faculty Mannheim, Heidelberg University, 68167 Mannheim, Germany; 3 Department of Cardiology and Angiology, Hannover Medical School, 30625 Hannover, Germany; 4 Department of Cardiology and Angiology II, University Heart Center Freiburg, 79189 Bad Krozingen, Germany; 5 Department of Internal Medicine and Cardiology, Mediclin Heart Centre Lahr, 77933 Lahr, Germany

**Keywords:** rotational atherectomy, percutaneous coronary intervention, coronary artery disease, sex

## Abstract

Present research on the influence of gender on the treatment of coronary artery disease (CAD) and the outcome after percutaneous coronary intervention (PCI) is inconsistent. Sex differences in the presentation of CAD and the success after treatment have been described. We intend to compare the male and female sex in the procedure and the long-term outcome of Rotational Atherectomy (RA). A total of 597 consecutive patients (20.3% female and 79.7% male, mean age 75.3 ± 8.9 years vs. 72.7 ± 9 years, *p* < 0.001) undergoing Rotational Atherectomy between 2015 and 2020 were enrolled in the analysis. Demographic and clinical data were registered. In-hospital, 1-year, and 3-year MACCEs (major adverse cardiac and cerebrovascular events) were calculated. Women presented more often with myocardial infarction (23.9% vs. 14.9%, *p* = 0.017). The intervention was mainly performed via femoral access compared to radial access (65.4% vs. 33.6%, *p* = 0.002). Women had a smaller diameter of the balloon predilatation compared to men (2.8 ± 0.5 mm vs. 3.15 ± 2.4 mm, *p* < 0.05) and a smaller maximum diameter of the implanted stent (3.5 ± 1.2 mm vs. 4.10 ± 6.5 mm, *p* = 0.01). In-hospital, 1-year-, and 3-year MACCEs did not differ between the sexes. After a multivariate analysis, no difference between men and women could be detected. In conclusion, this analysis shows differences between women and men in periprocedural characteristics but does not show any differences after RA regarding in-hospital, 1-year-, and 3-year MACCEs.

## 1. Introduction

Percutaneous coronary intervention (PCI) is a common treatment option for revascularization in patients with coronary artery disease (CAD) [1]. Depending on the severity of the coronary calcification, it may be necessary to first modify the coronary lesion to enable an optimal stent expansion. Rotational Atherectomy (RA) is an established procedure to abrade the plaque within the coronary vessel and make it permeable to the balloon catheter. Although routine use of RA has not shown a benefit on the outcome, its selected use is reasonable when penetration in the vessel is not possible or successful stenting is not expected due to the extent of calcification [2].

The randomized trial PREPARE-CALC compared RA with scoring or cutting balloons, both of which are strategies for the preparation of calcification. In complex coronary lesions which are classified as type C lesions, the intervention with RA before stent implantation has been shown to be superior to the use of scoring or cutting balloons [3].

PCI in patients with a greater extent of coronary calcification shows a higher rate of complications summarized as major adverse cardiac events (MACEs) compared to patients without coronary calcification [4]. In that study, MACEs included death, myocardial infarction, revascularization of the target lesion, and re-hospitalization for cardiac ischemia events.

Previous studies have also observed differences between men and women in the rate of complications associated with RA treatment, but the results vary from each other. The findings of Ford et al. showed that women seem to be at higher risk for adverse intraoperative events such as coronary dissection, cardiac tamponade, and bleeding, while the female sex was not independently related to long-term survival [5]. In contrast, Bouisset et al. found an independent association of female gender with a higher MACE rate, but no significant differences in procedural complications [6]. It is important to evaluate these results properly.

If it proves to be true that the female sex is a predictor for a worse outcome of RA, therapeutic consequences in the treatment of men and women should be considered. Therefore, we want to verify these previous results and find out whether our investigations lead to similar conclusions.

## 2. Materials and Methods

### 2.1. Study Population

In this retrospective observational study, we included consecutive adult patients who underwent RA PCI at our center between January 2015 and December 2020. A total of 597 patients were enrolled in this study. All procedures were carried out by experienced operators. PCI was performed for the following indications: inducible myocardial ischemia evaluated by stress echocardiography or myocardial perfusion imaging, or the presence of angina pectoris or angina equivalences. This study was approved by the local ethics committee (ethical approval number: EK 21-1100).

### 2.2. Procedural Characteristics

The complexity of lesions was classified as type A, B1, B2, and C lesions according to the American College of Cardiology/American Heart Association Lesion Classification (ACC/AHA) [7]. Severe calcification of the target lesion was defined by cineangiography (i.e., radiopacities noted without cardiac motion before contrast injection generally compromising both sides of the arterial lumen) [8]. The decision to perform RA was at the operators’ discretion and RA was performed initially due to heavy calcification in the target lesion or in previously failed PCI attempts due to undilatable or even uncrossable calcified lesions. All RA PCIs were performed by high-volume PCI operators (i.e., defined by a minimum of *n* = 50 RAs performed each year) using the ROTA-Rotational Atherectomy system and since September 2018 the ROTAPRO-RAS (Boston Scientific Corp., Natick, MA). Vascular access, burr size, and ablation speed were left to the operators’ discretion. Postinterventional 12-lead electrocardiograms were documented 24 h after PCI. 

### 2.3. Clinical Measures and Follow-Up

Medical histories, current medications, and electrocardiography were collected at study enrollment. Routine laboratory parameters were analyzed from venous blood samples according to the local laboratory’s standard procedure. Blood draws were taken at baseline and at 6, 8, and 24 h after the procedure. Troponin levels were measured at each time point. All patient data were collected from a standardized follow-up protocol, hospital admission records, the referring physician, or the outpatient clinic and were entered into a dedicated clinical database followed by outpatient visits or telephone contacts. We defined the primary endpoint as the rate of major adverse cardiac and cerebrovascular events (MACCEs) during a 3-year follow-up. MACCEs include mortality, myocardial infarction, target vessel revascularization (TVR), target lesion revascularization (TLR), and stroke. Our secondary study endpoint is the rate of in-hospital MACCEs.

### 2.4. Statistical Methods

The continuous data are presented as mean ± standard deviation or median (interquartile range, IQR) unless otherwise specified and were compared using the Student *t*-test. Wilcoxon rank-sum test and the Kruskal–Wallis test were applied for non-parametric continuous variables, as appropriate. Categorical variables were expressed as percentages and were compared using Pearson’s chi-square test or Fisher’s exact test. Multivariable analyses were calculated at baseline for the prediction of all-cause MACCEs by using Cox regression with backward elimination. Crude and adjusted hazard ratios with 95% confidence intervals (95% CI) were calculated after the selection of the confounding variables based on the grounds of univariable association with the given endpoints in the present study (*p* < 0.05). Cumulative event rates were calculated according to the Kaplan–Meier method, and comparisons were performed with the log-rank test. A *p* value of <0.05 was considered statistically significant, and all *p* values were 2-sided. All statistical analyses were performed with JMP 13.0 (SAS, Cary, NC, USA).

## 3. Results

RA PCI was performed in 597 patients, of whom 121 (20.3%) were female and 476 (79.7%) were male. The mean age of the male patients was 72.2 ± 9 years, while women were significantly older with an average age of 75.3 ± 8.9 years (*p* < 0.001).

There were no significant differences between men and women in baseline characteristics such as hypertension, hyperlipidemia, diabetes, smoking or previous smoker, or previous myocardial infarction. Nevertheless, the rate of acute myocardial infarction (MI) was significantly higher in women than in men (23.9% vs. 14.9%, *p* = 0.017). Differences in the existence of a previous coronary artery bypass graft (CABG) were not significant (*p* = 0.059), but an increased occurrence in men can be observed (31.9% vs. 22.7%). The estimated glomerular filtration rate (eGFR) pre-Rotablation was 68 ± 69 in women and 74 ± 22 in men (*p* = 0.067). Gender-related baseline characteristics are shown in Table 1. 

### 3.1. Procedural Characteristics

Femoral access was chosen more often than radial access (65.4% vs. 33.6%, *p* = 0.002). The diameter of the balloon predilatation was significantly smaller in women (2.8 ± 0.5 mm vs. 3.15 ± 2.4 mm, *p* < 0.05). The maximum inflation pressure predilatation was comparable in both groups with around 20.8 atm in women and 21.9 atm in men (*p* = 0.6). The number of implanted stents was similar (1.87 in men, 1.78 in women, *p* = 0.11). However, the maximum diameter of the stents differed significantly between the sexes. Females had a smaller maximum stent diameter of 3.5 ± 1.2 mm compared to males with a maximum stent diameter of 4.10 ± 6.5 mm (*p* = 0.01). The diameter of the balloon postdilatation showed no significant difference between men and women (3.97 ± 1.46 mm vs. 4.0 ± 0.68 mm, *p* = 0.82).

The most frequently used burr size was 1.5 mm (42.21%). Burr sizes used were well distributed in both groups. A burr size 2.0 mm was chosen in 4.69% of the patients and a size of 1.75 mm in 29.31%. The smallest burr in our study measured 1.25 mm and was only selected in 23.78% of the patients (Table 2).

### 3.2. Clinical Outcomes

Procedural complications such as perforation and pericardiocentesis did not differ between men and women. In-hospital MACCEs, our secondary endpoint, occurred in 3.3% of all patients. Women were affected more frequently than men (5.8% vs. 2.7%, *p* = 0.095), however, without statistical significance. A total of 15 patients died in the hospital (2.5%), 10 of them men (2.1%) and 5 women (4.1%). The estimated glomerular filtration rate (eGFR) post-intervention was significantly lower in females (63.9 ± 21 vs. 70.6 ± 22, *p* = 0.002). 

The follow-up period of our patients was up to 3 years. The 1-year MACCEs showed non-significant differences between males and females. They affected one-fifth (21.8%) of all patients. The most common events were the need for target vessel revascularization (TVR) (20.4%) and target lesion revascularization (TLR) (18.6%). TVR was required in 100 men (21%) and 22 women (18.2%; *p* = 0.491). TLR affected 90 men (18.9%) and 21 women (17.3%; *p* = 0.695).

**Figure 1:**
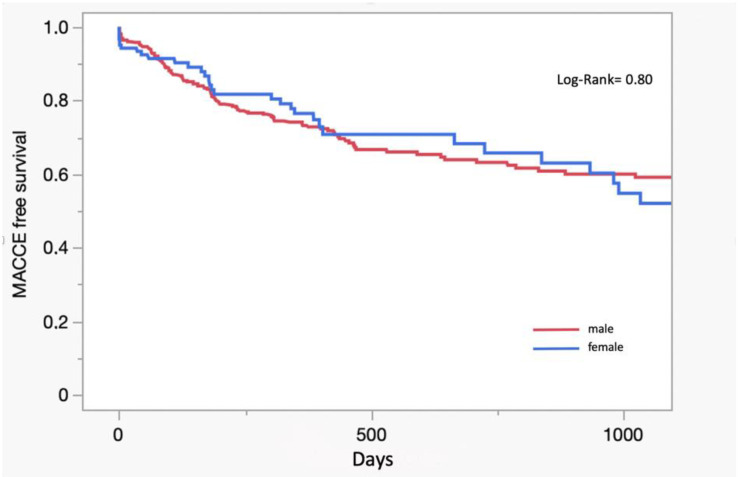
Kaplan–Meier curve.

A total of 44 patients (7.4%) passed away during the first year, of which 10.7% were female and 6.5% were male (Table 3). The Kaplan–Meier MACCE-free survival curve shows no significant difference between men and women (log-rank 0.80) (Figure 1).

The primary endpoint, defined as MACCEs within a 3-year follow-up, occurred in 155 patients (25.96%). It affected significantly more women than men (26.45% vs. 25.84%; *p* = 0.018). Three-year mortality was significantly higher in the female group compared to the male group (15.7% vs. 8.82%, *p* = 0.025) (Table 3). However, after adjustment of the data, gender was no longer a significant predictor for MACCEs. ACS was measured after an adjustment predictor for MACCEs, mortality (HR 2.33, 95% CI 1.58–3.43, *p* < 0.001), and for myocardial infarction (HR 5.25, 95% CI 1.29–21.34, *p* = 0.02) (Table 4).

## 4. Discussion

With this study, we aimed to analyze whether there is a difference between men and women regarding the implementation and success of RA. 

The main observations of our investigation are the following: (a) in a period of 3 years after the performance of RA, the female gender is not independently associated with a higher rate of MACCEs, and (b) the in-hospital outcome is comparable between men and women.

In the basic characteristics of our patients, it is striking that women were significantly older than men. This observation is consistent with prior studies comparing the genders in the RA treatment field [5,6,9]. There are several conceivable causes. Firstly, ACS in women often presents with atypical symptoms, making it difficult to identify and leading to a later medical consultation [10,11,12]. In addition, studies have shown a restrained use of revascularization therapy in women and a more frequent non-invasive treatment [13,14]. It is possible that the decision for an invasive therapy was therefore made at a later stage when other options had been exhausted.

Women presented significantly more often than men with ACS, which has also been noticed in previous studies [5,15]. Both men and women had slightly lower eGFRs after Rotablation than before the procedure. Among the men, the GFR fell from 74 to 70.6 mL/min. For the women, it fell from 68 to 63.9 mL/min. This is most likely due to the contrast media needed for PCI, as observed in other studies [16,17]. After the Rotablation, women had a significantly lower eGFRs than men. As documented in many studies, women often suffer from more comorbidities than men, including poorer renal function [6,10,18]. 

Age and comorbidities are risk factors for complications and mortality after PCI [19,20]. Studies disagree on the role of gender as a risk factor for procedural complications and the outcome of coronary interventions [5,6]. For complications associated with RA, we did not find a difference between men and women in the events of bleeding, perforation, or pericardiocentesis. In our study, the early appearance of MACCEs in-hospital and after 1 year also did not differ between the sexes. However, at the 3-year follow-up, women had significantly more MACCEs than men.

After adjustment, sex has not been proven to be a predictor for MACCEs. This suggests that confounding factors have influenced the result. Women were older than men, but age did also not show significance as a predictor for mortality. In fact, a significant predictor for MACCEs has turned out to be ACS. Since the women in our study were reported to have ACS much more often, this is likely a reason for the correlation of the female gender with the occurrence of MACCEs.

All in all, gender itself has not proven to be an independent predictor for MACCEs or mortality after RA.

Regarding the long-time MACCE-free survival, our findings are largely in line with the result of prior studies, which also found significantly higher MACCE rates in women that, as in our case, were not independently related after adjustment [5,9].

## 5. Limitations

Our study has several limitations. First of all, it is based on a retrospective non-randomized registry within a single-center study, where a large cohort of patients underwent elective PCI by experienced operators. Furthermore, the indication for RA PCI and the selected device was left to the operator’s discretion and may have varied depending upon their technical expertise and experience. 

Our study included an unequal number of female to male patients. With a share of 20.3%, women were under-represented compared to men. The two groups also differed in age.

More studies with a larger number of participants are needed to confirm our results. 

## 6. Conclusions

Women were older, presented with more incidences of ACS, and had a higher risk of MACCEs in a 3-year-follow-up. After adjustment, the female sex has not been confirmed to be an independent risk factor for the rate of MACCEs after RA PCI. We did not find gender differences in periprocedural complications.

## Figures and Tables

**Table 1:** Gender-related baseline characteristics.

Patient Characteristics	Total Number	Women	Men	*p* Value
	(*n* = 597)	(*n* = 121)	(*n* = 476)	
Age (years)	73.3 ± 9	75.3 ± 8.9	72.2 ± 9	<0.001
Hypertension	532 (92.3%)	107 (92.2%)	425 (92.4%)	0.956
Hypercholesterinemia	503 (89.9%)	95 (86.3%)	408 (90.8%)	0.158
Diabetes mellitus	209 (37.6%)	34 (30.9%)	175 (39.2%)	0.106
Smoker or previous smoker	58 (10.5%)	13 (11.8%)	45 (10.1%)	0.611
Previous MI	184 (34.5%)	37 (33.6%)	147 (34.7%)	0.826
Acute MI	100 (16.7%)	29 (23.9%)	71 (14.9%)	0.017
Previous CABG	164 (30%)	25 (22.7%)	139 (31.9%)	0.059
eGFR pre-Rota	71 ± 11	68 ± 11	74 ± 22	0.067
LVEF (%)				0.771
>51%	318 (59.2%)	69 (61%)	259 (58.7%)	
41–51%	126 (23.4%)	25 (22.1%)	101 (23.8%)	
30–40%	65 (12.1%)	15 (13.3%)	50 (11.8%)	
0–29%	28 (5.2%)	4 (3.5%)	24 (5.6%)	

eGFR = estimated glomerular filtration rate, CABG = coronary artery bypass graft, MI = myocardial infarction, LVEF = left ventricular ejection fraction.

**Table 2:** Technical characteristics.

Overall Procedural Results	Total Number (*n* = 597)	Women (*n* = 121)	Men (*n* = 476)	*p* Value
Balloon diameter predilatation, mm		2.8 ± 0.5	3.15 ± 2.4	<0.05
Maximum inflation pressure predilatation, atm		20.8 ± 6.2	21.94 ± 13.0	0.6
Pre-Rota stenosis, %		87.9% ± 14.3	87.47% ± 14.8	0.92
Post-stent residual stenosis, %		1.42 ± 9.5	2.7 ± 13.5	0.84
Number of stents implanted		1.78 ± 1.0	1.87 ± 1.1	0.11
Diameter of implanted stent, max., mm		3.5 ± 1.2	4.10 ± 6.5	0.01
Overall stent length, mm		50.3 ± 30.0	53.3 ± 32.0	0.334
Balloon diameter postdilatation, mm		4.0 ± 0.68	3.97 ± 1.46	0.82
Postdilatation pressure, atm		21.11 ± 4.65	21.66 ± 5.74	0.52
Burr size used				0.474
1.25 mm	142 (23.78%)	26 (21.48%)	116 (24.36%)	
1.50 mm	252 (42.21%)	52 (42.97%)	200 (42.01%)	
1.75 mm	175 (29.31%)	34 (28.09%)	141 (29.62%)	
2.00 mm	28 (4.69%)	9 (7.43%)	19 (3.99%)	
Access site				0.002
Radial access	192 (33.6%)	23 (11.98%)	169 (88.02%)	
Femoral access	374 (65.4%)	90 (24.06%)	284 (75.94%)	

**Table 3:** Clinical outcomes.

Clinical Outcomes	All Patients (*n* = 597)	Women (*n* = 121)	Men (*n* = 476)	*p* Value
In-hospital MACCEs	20 (3.3%)	7 (5.8%)	13 (2.7%)	0.095
In-hospital Mortality	15 (2.5%)	5 (4.1%)	10 (2.1%)	0.202
In-hospital MI	4 (0.67%)	0 (0%)	4 (0.8%)	0.311
In-hospital TVR	20 (3.3%)	7 (5.8%)	13 (2.7%)	0.095
In-hospital Stroke	1 (0.17%)	1 (0.8%)	0 (0%)	0.202
In-hospital TLR	20 (3.3%)	7 (5.8%)	13 (2.7%)	0.095
Perforation	28 (4.7%)	8 (6.6%)	20 (4.3%)	0.281
Pericardiocentesis	8 (1.3%)	3 (2.5%)	5 (1%)	0.207
Bleeding	57 (9.5%)	7 (5.8%)	50 (10.5%)	0.09
eGFR max. post-Rota	68.6 ± 21	63.9 ± 21	70.6 ± 22	0.002
1-year MACCEs	130 (21.8%)	24 (19.8%)	106 (22.3%)	0.562
1-year Mortality	44 (7.4%)	13 (10.7)	31 (6.5%)	0.111
1-year MI	10 (1.7%)	2 (1.6%)	8 (1.7%)	0.983
1-year TVR	122 (20.4%)	22 (18.2%)	100 (21%)	0.491
1-year Stroke	4 (0.7%)	2 (1.6%)	2 (0.4%)	0.137
1-year TLR	111 (18.6%)	21 (17.3%)	90 (18.9%)	0.695
3-year MACCEs	155 (25.96%)	32 (26.45%)	123 (25.84%)	0.018
3-year Mortality	61 (10.22%)	19 (15.70%)	42 (8.82%)	0.025
3-year MI	12 (2%)	3 (2.48%)	9 (1.89%)	0.716
3-year TVR	149 (24.96%)	30 (24.79%)	119 (25%)	0.962
3-year Stroke	9 (1.51%)	4 (3.31%)	5 (1.05%)	0.087
3-year TLR	138 (23.12%)	29 (23.97%)	109 (22.90%)	0.803

eGFR = estimated glomerular filtration rate, MACCEs = major adverse cardiac and cerebrovascular events, MI = myocardial infarction, TLR = target lesion revascularization, TVR = target vessel revascularization.

**Table 4:** Univariate and multivariate predictors of 3-year MACCEs in total cohort assessed by Cox regression analysis.

**a: Cox Regression Analysis for Predictors of MACCEs**						
	**Univariate Analysis**			**Multivariate Analysis**		
	**HR**	**95% CI**	***p*** **Value**	**HR**	**95% CI**	***p*** **Value**
Age (change per year)	1.004	0.99–1.02	0.67	0.99	0.98–1.02	0.76
Gender (female)	1.05	0.71–1.54	0.81	0.95	0.62–1.46	0.80
ACS	2.22	1.58–3.13	<0.001	2.33	1.58–3.43	<0.001
Diabetes mellitus	1.09	0.78–1.53	0.63	0.98	0.89–1.40	0.92
History of CABG	1.14	0.80–1.63	0.47	1.16	0.8–1.67	0.44
**b: Cox Regression Analysis for Predictors Of All-Cause Mortality**						
	**Univariate Analysis**			**Multivariate Analysis**		
	**HR**	**95% CI**	***p*** **Value**	**HR**	**95% CI**	***p*** **Value**
Age (change per year)	1.06	1.03–1.10	<0.001	1.04	1.01–1.07	0.03
Gender (female)	1.82	1.07–3.10	0.03	1.42	0.79–2.56	0.24
ACS	5.10	3.11–8.37	<0.001	2.33	1.58–3.43	<0.001
Diabetes mellitus	1.27	0.76–2.14	0.37	1.22	0.73–2.22	0.47
History of CABG	1.06	0.61–1.85	0.84	1.27	0.8–1.67	0.40
**c: Cox Regression Analysis for Predictors of Myocardial Infarction**						
	**Univariate Analysis**			**Multivariate Analysis**		
	**HR**	**95% CI**	***p*** **Value**	**HR**	**95% CI**	***p*** **Value**
Age (change per year)	1.005	0.94–1.07	0.90	0.98	0.91–1.06	0.61
Gender (female)	1.51	0.4 1–5.69	0.54	1.11	0.22–5.61	0.90
ACS	4.76	1.43–15.87	0.01	5.25	1.29–21.34	0.02
Diabetes mellitus	2.42	0.64–9.22	0.20	2.18	0.56–8.56	0.26
History of CABG	1.05	0.26–4.21	0.95	1.22	0.30–4.98	0.78

ACS = acute coronary syndrome, CABG = coronary artery bypass graft.

## Data Availability

The datasets used and/or analyzed during the current study are available from the corresponding author on reasonable request.
